# 4-Hydrazinyl-1-isobutyl-1*H*-imidazo[4,5-*c*]quinoline

**DOI:** 10.1107/S1600536811001553

**Published:** 2011-01-15

**Authors:** Wan-Sin Loh, Hoong-Kun Fun, Reshma Kayarmar, S. Viveka, G. K. Nagaraja

**Affiliations:** a X-ray Crystallography Unit, School of Physics, Universiti Sains Malaysia, 11800 USM, Penang, Malaysia; b Department of Chemistry, Mangalore University, Karnataka, India

## Abstract

In the title compound, C_14_H_17_N_5_, the 1*H*-imidazo[4,5-*c*]quinoline ring system is essentially planar, with a maximum deviation of 0.0325 (7) Å. In the crystal, a pair of inter­molecular N—H⋯N hydrogen bonds link neighbouring mol­ecules, forming an inversion dimer and generate an *R*_2_^2^(10) ring motif. These dimers are further connected into a chain along the *b* axis *via* inter­molecular C—H⋯N hydrogen bonds, resulting in an *R*_2_^2^(14) ring motif.

## Related literature

For background to quinolines and their microbial activity, see: Roth & Fenner (2000 ▶); Miller *et al.* (1999 ▶); Hirota *et al.* (2002 ▶). For bond-length data, see: Allen *et al.* (1987 ▶). For a related structure, see: Loh *et al.* (2011 ▶). For hydrogen-bond motifs, see: Bernstein *et al.* (1995 ▶). For the stability of the temperature controller used in the data collection, see: Cosier & Glazer (1986 ▶).
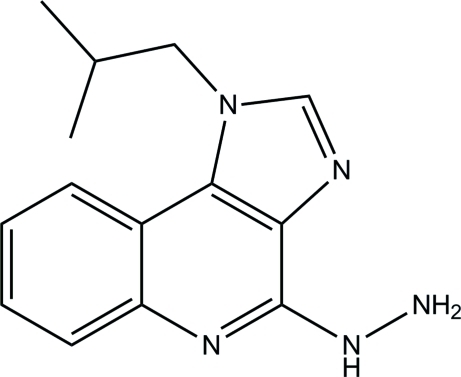


## Experimental

### 

#### Crystal data

C_14_H_17_N_5_
*M* *_r_* = 255.33
Triclinic, 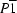

*a* = 5.4735 (2) Å
*b* = 9.1275 (3) Å
*c* = 13.3814 (5) Å
α = 98.076 (1)°
β = 101.787 (1)°
γ = 96.269 (1)°
*V* = 641.35 (4) Å^3^
*Z* = 2
Mo *K*α radiation
μ = 0.08 mm^−1^
*T* = 100 K
0.68 × 0.42 × 0.09 mm

#### Data collection

Bruker SMART APEXII DUO CCD area-detector diffractometer
Absorption correction: multi-scan (*SADABS*; Bruker, 2009 ▶) *T*_min_ = 0.945, *T*_max_ = 0.992
20646 measured reflections
5797 independent reflections
4836 reflections with *I* > 2σ(*I*)
*R*_int_ = 0.023

#### Refinement

*R*[*F* ^2^ > 2σ(*F* ^2^)] = 0.040
*wR*(*F* ^2^) = 0.137
*S* = 1.12
5797 reflections
240 parameters
H atoms treated by a mixture of independent and constrained refinement
Δρ_max_ = 0.53 e Å^−3^
Δρ_min_ = −0.32 e Å^−3^

### 

Data collection: *APEX2* (Bruker, 2009 ▶); cell refinement: *SAINT* (Bruker, 2009 ▶); data reduction: *SAINT*; program(s) used to solve structure: *SHELXTL* (Sheldrick, 2008 ▶); program(s) used to refine structure: *SHELXTL*; molecular graphics: *SHELXTL*; software used to prepare material for publication: *SHELXTL* and *PLATON* (Spek, 2009 ▶).

## Supplementary Material

Crystal structure: contains datablocks global, I. DOI: 10.1107/S1600536811001553/is2657sup1.cif

Structure factors: contains datablocks I. DOI: 10.1107/S1600536811001553/is2657Isup2.hkl

Additional supplementary materials:  crystallographic information; 3D view; checkCIF report

## Figures and Tables

**Table 1:** Hydrogen-bond geometry (Å, °)

*D*—H⋯*A*	*D*—H	H⋯*A*	*D*⋯*A*	*D*—H⋯*A*
N4—H1*N*4⋯N3^i^	0.883 (16)	2.130 (15)	2.9429 (9)	152.9 (15)
C5—H5⋯N5^ii^	1.012 (12)	2.437 (11)	3.3700 (10)	152.9 (10)

Symmetry codes: (i) 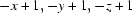; (ii) 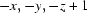.

**Fig. 1.**
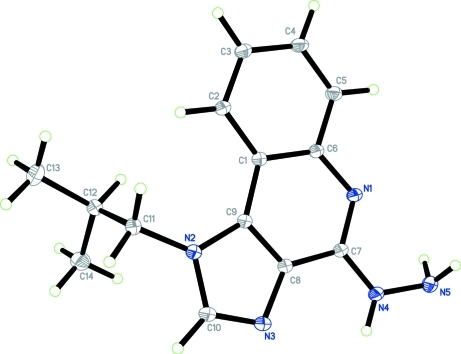
The molecular structure of the title compound, showing 50% probability displacement ellipsoids and the atom-numbering scheme.

**Fig. 2.**
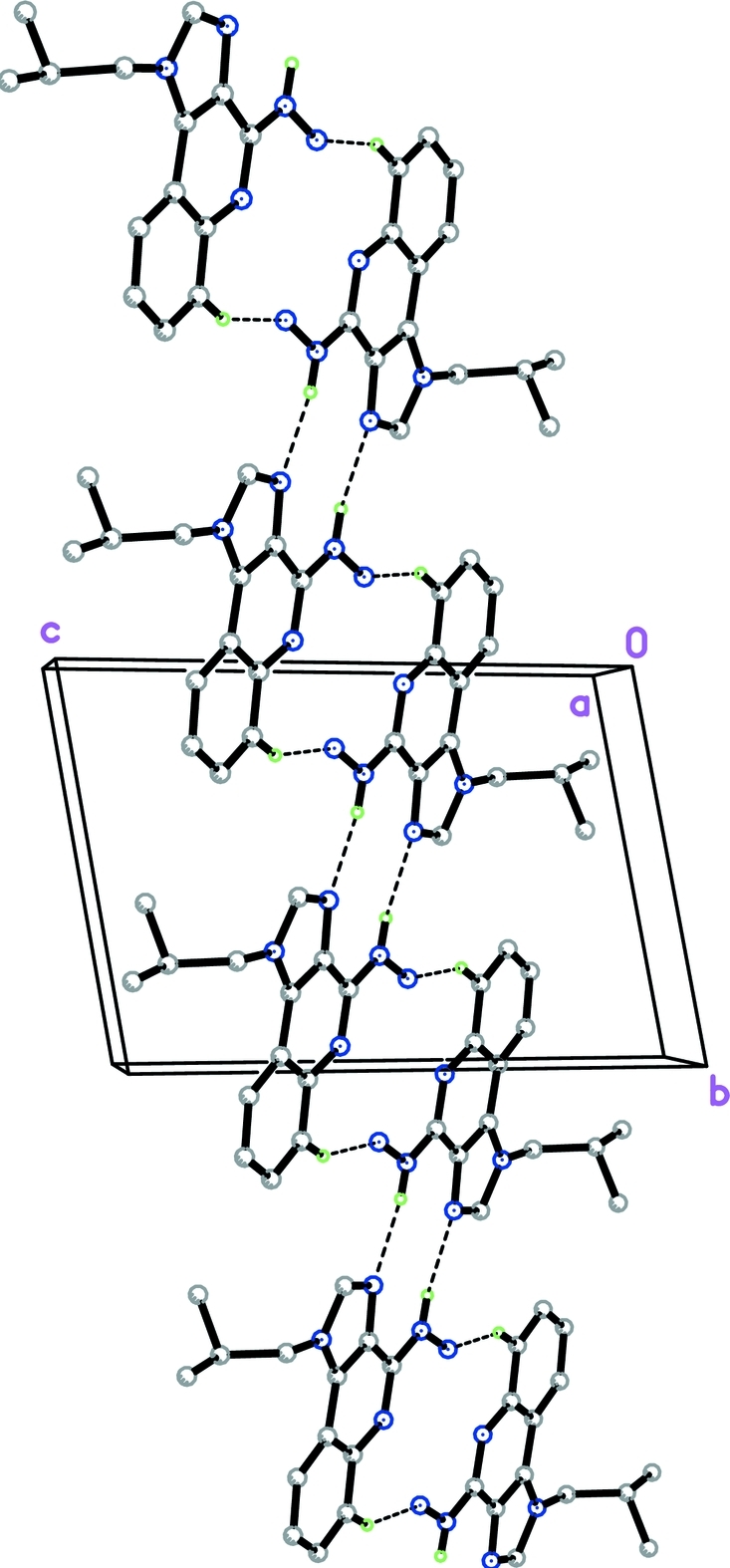
The crystal packing of the title compound, showing the chains along the b axis. H atoms not involved in the intermolecular interactions (dashed lines) have been omitted for clarity.

## supplementary crystallographic information

### Comment

The quinoline scaffold is present in many classes of biologically active compounds (Roth & Fenner, 2000), as for example, in 1*H*-imidazo-[4,5-*c*]quinolines that induce IFN, as well as other cytokines, in mice, rats, guinea pigs, monkeys and humans (Miller *et al.*, 1999). This initiated the syntheses of a series of compounds with differing substitution at N-1, C-2, C-4 and on substitution on the benzene ring. Phenoxymethyl and benzyl groups at C-2 increase the activity. All other C-4 substituents investigated fail to induce IFN production. This investigation encouraged us to substitute C-4 by- NHNH_2_ in continuation of our research to explore novel series of immune response modifiers in an effort to find small molecules that treat diseases involving the immune system (Hirota *et al.*, 2002).

In the title compound (Fig. 1), the 1*H*-imidazo[4,5-*c*]quinoline ring (C1–C6/N1/C7/C8/N3/C10/N2/C9) is approximately planar with a maximum deviation of 0.0325 (7) Å at atom C1. The torsion angle formed between this ring system and the isobutyl moiety, C10–N2–C11–C12, is 101.17 (8)°. Bond lengths (Allen *et al.*, 1987) and angles are within the normal ranges and are comparable to the related structure (Loh *et al.*, 2011).

In the crystal packing (Fig. 2), intermolecular N4—H1N4···N3 hydrogen bonds (Table 1) link the neighbouring molecules to form dimers and generate *R*_2_^2^(10) ring motifs (Bernstein *et al.*, 1995). These dimers are further connected into chains down the *b* axis *via* intermolecular C5—H5···N5 hydrogen bonds (Table 1), resulting in *R*_2_^2^(14) ring motifs (Bernstein *et al.*, 1995).

### Experimental

4-Chloro-1-(2-methylpropyl)-1*H*-imidazo[4,5-*c*]quinolone (10 g, 0.0385 mole) and hydrazine-hydrate (80%, 19.3 g, 0.385 mole) in ethanol was refluxed for 9 h during which white solids separated out. After cooling to room temperature, the resulting 4-hydrazinyl-1-(2-methylpropyl)-1*H*-imidazo[4,5-*c*]quinoline was filtered off, dried and crystallized from ethanol. Yield, 7.4 g (74%). Crystals suitable for X-ray analysis were obtained from ethanol by slow evaporation.

### Refinement

All H atoms were located from difference Fourier map and were refined freely [N—H = 0.883 (15) to 0.909 (14) Å; C—H = 0.978 (13) to 1.037 (12) Å].

### Crystal data

**Table:**

C_14_H_17_N_5_	*Z* = 2
*M**_r_* = 255.33	*F*(000) = 272
Triclinic, *P*1	*D*_x_ = 1.322 Mg m^−^^3^
Hall symbol: -P 1	Mo *K*α radiation, λ = 0.71073 Å
*a* = 5.4735 (2) Å	Cell parameters from 9851 reflections
*b* = 9.1275 (3) Å	θ = 2.5–35.6°
*c* = 13.3814 (5) Å	µ = 0.08 mm^−^^1^
α = 98.076 (1)°	*T* = 100 K
β = 101.787 (1)°	Plate, yellow
γ = 96.269 (1)°	0.68 × 0.42 × 0.09 mm
*V* = 641.35 (4) Å^3^	

### Data collection

**Table:**

Bruker SMART APEXII DUO CCD area-detector diffractometer	5797 independent reflections
Radiation source: fine-focus sealed tube	4836 reflections with *I* > 2σ(*I*)
graphite	*R*_int_ = 0.023
φ and ω scans	θ_max_ = 35.6°, θ_min_ = 1.6°
Absorption correction: multi-scan (*SADABS*; Bruker, 2009)	*h* = −8→8
*T*_min_ = 0.945, *T*_max_ = 0.992	*k* = −14→14
20646 measured reflections	*l* = −21→21

### Refinement

**Table:**

Refinement on *F*^2^	Primary atom site location: structure-invariant direct methods
Least-squares matrix: full	Secondary atom site location: difference Fourier map
*R*[*F*^2^ > 2σ(*F*^2^)] = 0.040	Hydrogen site location: inferred from neighbouring sites
*wR*(*F*^2^) = 0.137	H atoms treated by a mixture of independent and constrained refinement
*S* = 1.12	*w* = 1/[σ^2^(*F*_o_^2^) + (0.0821*P*)^2^ + 0.0687*P*] where *P* = (*F*_o_^2^ + 2*F*_c_^2^)/3
5797 reflections	(Δ/σ)_max_ = 0.001
240 parameters	Δρ_max_ = 0.53 e Å^−^^3^
0 restraints	Δρ_min_ = −0.32 e Å^−^^3^

### Special details

**Table:**

Experimental. The crystal was placed in the cold stream of an Oxford Cryosystems Cobra open-flow nitrogen cryostat (Cosier & Glazer, 1986) operating at 100.0 (1) K.
Geometry. All e.s.d.'s (except the e.s.d. in the dihedral angle between two l.s. planes) are estimated using the full covariance matrix. The cell e.s.d.'s are taken into account individually in the estimation of e.s.d.'s in distances, angles and torsion angles; correlations between e.s.d.'s in cell parameters are only used when they are defined by crystal symmetry. An approximate (isotropic) treatment of cell e.s.d.'s is used for estimating e.s.d.'s involving l.s. planes.
Refinement. Refinement of *F*^2^ against ALL reflections. The weighted *R*-factor *wR* and goodness of fit *S* are based on *F*^2^, conventional *R*-factors *R* are based on *F*, with *F* set to zero for negative *F*^2^. The threshold expression of *F*^2^ > σ(*F*^2^) is used only for calculating *R*-factors(gt) *etc*. and is not relevant to the choice of reflections for refinement. *R*-factors based on *F*^2^ are statistically about twice as large as those based on *F*, and *R*- factors based on ALL data will be even larger.

### Fractional atomic coordinates and isotropic or equivalent isotropic displacement parameters (Å^2^)

**Table:**

	*x*	*y*	*z*	*U*_iso_*/*U*_eq_	
N3	0.66263 (12)	0.41343 (6)	0.40530 (5)	0.01739 (12)	
N2	0.83913 (11)	0.28950 (6)	0.28779 (4)	0.01494 (11)	
N1	0.29129 (11)	0.04458 (6)	0.39588 (4)	0.01382 (11)	
N4	0.27467 (12)	0.27155 (7)	0.49606 (5)	0.01719 (12)	
N5	0.09896 (12)	0.21044 (7)	0.54799 (5)	0.01683 (11)	
C9	0.66447 (12)	0.18519 (7)	0.31040 (5)	0.01294 (11)	
C1	0.58776 (12)	0.02794 (7)	0.27674 (5)	0.01284 (11)	
C2	0.68633 (13)	−0.06582 (7)	0.20610 (5)	0.01545 (12)	
C3	0.60210 (14)	−0.21733 (7)	0.18278 (5)	0.01727 (13)	
C4	0.41445 (14)	−0.27919 (7)	0.22854 (6)	0.01769 (13)	
C5	0.31322 (13)	−0.19005 (7)	0.29698 (5)	0.01611 (12)	
C6	0.39780 (12)	−0.03437 (7)	0.32402 (5)	0.01301 (11)	
C7	0.37122 (12)	0.18938 (7)	0.42576 (5)	0.01334 (11)	
C8	0.55945 (13)	0.26428 (7)	0.38309 (5)	0.01385 (11)	
C10	0.82902 (15)	0.42279 (7)	0.34703 (5)	0.01813 (13)	
C11	1.00391 (12)	0.26889 (7)	0.21544 (5)	0.01498 (12)	
C12	0.86854 (13)	0.26095 (7)	0.10230 (5)	0.01536 (12)	
C13	1.04956 (16)	0.22187 (9)	0.03277 (6)	0.02337 (15)	
C14	0.77216 (15)	0.40809 (8)	0.08500 (6)	0.02100 (14)	
H12	0.714 (2)	0.1783 (13)	0.0858 (9)	0.022 (3)*	
H5	0.188 (2)	−0.2330 (14)	0.3351 (9)	0.023 (3)*	
H11A	1.082 (2)	0.1765 (14)	0.2249 (9)	0.021 (3)*	
H11B	1.135 (2)	0.3583 (12)	0.2340 (8)	0.016 (2)*	
H3	0.684 (3)	−0.2852 (15)	0.1362 (10)	0.030 (3)*	
H14A	0.647 (2)	0.4297 (15)	0.1284 (10)	0.028 (3)*	
H2	0.824 (3)	−0.0234 (15)	0.1744 (10)	0.028 (3)*	
H14B	0.684 (2)	0.4015 (15)	0.0128 (10)	0.027 (3)*	
H13A	1.198 (3)	0.3048 (16)	0.0476 (10)	0.035 (3)*	
H13B	0.967 (3)	0.2083 (16)	−0.0425 (11)	0.042 (4)*	
H14C	0.914 (3)	0.4902 (15)	0.1002 (10)	0.027 (3)*	
H2N5	−0.034 (3)	0.1553 (16)	0.5007 (11)	0.034 (3)*	
H1N5	0.168 (2)	0.1427 (14)	0.5847 (9)	0.026 (3)*	
H4	0.351 (3)	−0.3897 (15)	0.2119 (11)	0.033 (3)*	
H1N4	0.338 (3)	0.3668 (17)	0.5163 (11)	0.039 (4)*	
H10	0.947 (2)	0.5153 (13)	0.3482 (9)	0.023 (3)*	
H13C	1.111 (3)	0.1258 (16)	0.0456 (11)	0.036 (3)*	

### Atomic displacement parameters (Å^2^)

**Table:**

	*U*^11^	*U*^22^	*U*^33^	*U*^12^	*U*^13^	*U*^23^
N3	0.0241 (3)	0.0107 (2)	0.0180 (2)	−0.00015 (19)	0.0094 (2)	−0.00037 (18)
N2	0.0186 (2)	0.0106 (2)	0.0163 (2)	−0.00019 (17)	0.00804 (18)	0.00044 (17)
N1	0.0166 (2)	0.0107 (2)	0.0146 (2)	0.00162 (17)	0.00618 (18)	−0.00006 (16)
N4	0.0236 (3)	0.0116 (2)	0.0184 (2)	0.00107 (19)	0.0123 (2)	−0.00060 (18)
N5	0.0190 (3)	0.0158 (2)	0.0174 (2)	0.00174 (19)	0.00878 (19)	0.00190 (18)
C9	0.0160 (3)	0.0101 (2)	0.0132 (2)	0.00083 (19)	0.00551 (19)	0.00118 (17)
C1	0.0152 (3)	0.0103 (2)	0.0134 (2)	0.00142 (18)	0.00501 (19)	0.00076 (18)
C2	0.0191 (3)	0.0117 (2)	0.0168 (3)	0.0018 (2)	0.0085 (2)	0.00019 (19)
C3	0.0211 (3)	0.0121 (2)	0.0196 (3)	0.0017 (2)	0.0096 (2)	−0.0009 (2)
C4	0.0209 (3)	0.0107 (2)	0.0215 (3)	0.0000 (2)	0.0091 (2)	−0.0015 (2)
C5	0.0180 (3)	0.0112 (2)	0.0195 (3)	−0.0001 (2)	0.0082 (2)	−0.0001 (2)
C6	0.0145 (2)	0.0109 (2)	0.0140 (2)	0.00148 (18)	0.00531 (19)	0.00056 (18)
C7	0.0164 (3)	0.0112 (2)	0.0131 (2)	0.00201 (19)	0.00547 (19)	0.00073 (18)
C8	0.0180 (3)	0.0106 (2)	0.0135 (2)	0.00126 (19)	0.00616 (19)	0.00052 (18)
C10	0.0244 (3)	0.0108 (2)	0.0195 (3)	−0.0011 (2)	0.0096 (2)	−0.0006 (2)
C11	0.0157 (3)	0.0136 (2)	0.0166 (2)	0.0008 (2)	0.0067 (2)	0.00201 (19)
C12	0.0174 (3)	0.0133 (2)	0.0162 (2)	0.0012 (2)	0.0064 (2)	0.00193 (19)
C13	0.0275 (4)	0.0252 (3)	0.0210 (3)	0.0058 (3)	0.0129 (3)	0.0032 (2)
C14	0.0244 (3)	0.0170 (3)	0.0226 (3)	0.0049 (2)	0.0055 (2)	0.0048 (2)

### Geometric parameters (Å, °)

**Table:**

N3—C10	1.3179 (9)	C3—H3	1.020 (13)
N3—C8	1.3821 (8)	C4—C5	1.3798 (9)
N2—C10	1.3687 (9)	C4—H4	1.008 (14)
N2—C9	1.3828 (8)	C5—C6	1.4170 (9)
N2—C11	1.4590 (9)	C5—H5	1.011 (12)
N1—C7	1.3236 (8)	C7—C8	1.4322 (9)
N1—C6	1.3820 (8)	C10—H10	1.002 (12)
N4—C7	1.3484 (8)	C11—C12	1.5315 (9)
N4—N5	1.4085 (9)	C11—H11A	0.999 (12)
N4—H1N4	0.883 (15)	C11—H11B	0.993 (11)
N5—H2N5	0.909 (14)	C12—C14	1.5258 (10)
N5—H1N5	0.909 (13)	C12—C13	1.5282 (10)
C9—C8	1.3854 (9)	C12—H12	1.037 (12)
C9—C1	1.4314 (9)	C13—H13A	1.014 (14)
C1—C2	1.4138 (9)	C13—H13B	1.000 (14)
C1—C6	1.4302 (9)	C13—H13C	0.998 (14)
C2—C3	1.3795 (9)	C14—H14A	1.001 (13)
C2—H2	1.008 (14)	C14—H14B	0.978 (13)
C3—C4	1.4058 (10)	C14—H14C	0.985 (14)
			
C10—N3—C8	103.93 (5)	N1—C7—C8	121.10 (6)
C10—N2—C9	106.32 (6)	N4—C7—C8	117.90 (6)
C10—N2—C11	124.73 (6)	N3—C8—C9	111.27 (6)
C9—N2—C11	128.95 (5)	N3—C8—C7	128.47 (6)
C7—N1—C6	118.55 (6)	C9—C8—C7	120.25 (6)
C7—N4—N5	123.58 (6)	N3—C10—N2	113.44 (6)
C7—N4—H1N4	118.2 (10)	N3—C10—H10	124.7 (7)
N5—N4—H1N4	117.8 (10)	N2—C10—H10	121.7 (7)
N4—N5—H2N5	109.3 (9)	N2—C11—C12	113.35 (6)
N4—N5—H1N5	109.3 (8)	N2—C11—H11A	108.6 (7)
H2N5—N5—H1N5	104.1 (12)	C12—C11—H11A	110.6 (7)
N2—C9—C8	105.04 (5)	N2—C11—H11B	106.1 (6)
N2—C9—C1	134.08 (6)	C12—C11—H11B	107.6 (6)
C8—C9—C1	120.87 (6)	H11A—C11—H11B	110.5 (9)
C2—C1—C6	119.84 (6)	C14—C12—C13	111.16 (6)
C2—C1—C9	126.25 (6)	C14—C12—C11	110.90 (5)
C6—C1—C9	113.89 (6)	C13—C12—C11	108.94 (6)
C3—C2—C1	120.58 (6)	C14—C12—H12	107.7 (6)
C3—C2—H2	119.1 (7)	C13—C12—H12	110.1 (7)
C1—C2—H2	120.3 (7)	C11—C12—H12	108.0 (6)
C2—C3—C4	119.88 (6)	C12—C13—H13A	109.6 (8)
C2—C3—H3	120.0 (8)	C12—C13—H13B	112.4 (9)
C4—C3—H3	120.0 (8)	H13A—C13—H13B	108.1 (11)
C5—C4—C3	120.75 (6)	C12—C13—H13C	109.9 (8)
C5—C4—H4	118.6 (8)	H13A—C13—H13C	109.8 (12)
C3—C4—H4	120.6 (8)	H13B—C13—H13C	106.9 (12)
C4—C5—C6	120.97 (6)	C12—C14—H14A	110.3 (7)
C4—C5—H5	122.2 (7)	C12—C14—H14B	110.4 (8)
C6—C5—H5	116.6 (7)	H14A—C14—H14B	106.7 (10)
N1—C6—C5	116.69 (6)	C12—C14—H14C	110.4 (8)
N1—C6—C1	125.32 (6)	H14A—C14—H14C	111.1 (11)
C5—C6—C1	117.98 (6)	H14B—C14—H14C	107.9 (11)
N1—C7—N4	120.99 (6)		
			
C10—N2—C9—C8	−0.05 (7)	C6—N1—C7—N4	−179.65 (6)
C11—N2—C9—C8	−179.38 (6)	C6—N1—C7—C8	1.52 (10)
C10—N2—C9—C1	−178.69 (7)	N5—N4—C7—N1	4.70 (11)
C11—N2—C9—C1	1.98 (12)	N5—N4—C7—C8	−176.43 (6)
N2—C9—C1—C2	0.80 (12)	C10—N3—C8—C9	−0.50 (8)
C8—C9—C1—C2	−177.67 (6)	C10—N3—C8—C7	178.70 (7)
N2—C9—C1—C6	178.97 (7)	N2—C9—C8—N3	0.34 (8)
C8—C9—C1—C6	0.50 (9)	C1—C9—C8—N3	179.20 (6)
C6—C1—C2—C3	−0.43 (10)	N2—C9—C8—C7	−178.94 (6)
C9—C1—C2—C3	177.64 (6)	C1—C9—C8—C7	−0.07 (10)
C1—C2—C3—C4	0.77 (11)	N1—C7—C8—N3	179.87 (6)
C2—C3—C4—C5	−0.14 (11)	N4—C7—C8—N3	1.00 (11)
C3—C4—C5—C6	−0.84 (11)	N1—C7—C8—C9	−0.99 (10)
C7—N1—C6—C5	177.81 (6)	N4—C7—C8—C9	−179.86 (6)
C7—N1—C6—C1	−1.08 (10)	C8—N3—C10—N2	0.48 (8)
C4—C5—C6—N1	−177.81 (6)	C9—N2—C10—N3	−0.28 (8)
C4—C5—C6—C1	1.16 (10)	C11—N2—C10—N3	179.09 (6)
C2—C1—C6—N1	178.35 (6)	C10—N2—C11—C12	−101.17 (8)
C9—C1—C6—N1	0.05 (10)	C9—N2—C11—C12	78.06 (8)
C2—C1—C6—C5	−0.52 (10)	N2—C11—C12—C14	63.65 (7)
C9—C1—C6—C5	−178.82 (6)	N2—C11—C12—C13	−173.69 (6)

### Hydrogen-bond geometry (Å, °)

**Table:**

*D*—H···*A*	*D*—H	H···*A*	*D*···*A*	*D*—H···*A*
N4—H1N4···N3^i^	0.883 (16)	2.130 (15)	2.9429 (9)	152.9 (15)
C5—H5···N5^ii^	1.012 (12)	2.437 (11)	3.3700 (10)	152.9 (10)

Symmetry codes: (i) −*x*+1, −*y*+1, −*z*+1; (ii) −*x*, −*y*, −*z*+1.
